# Supplementary material for: New relevance and significance measures to replace p-values
Source: PLoS One. 2021 Jun 16;16(6):e0252991. doi: 10.1371/journal.pone.0252991 (PMC8208587; doi:10.1371/journal.pone.0252991)
Supplement: S1 Appendix — (PDF) [file pone.0252991.s001.pdf]

## S1 Appendix

**Derivation of** (11) **and** (12)

The general formula for the inversion of a partitioned matrix reads

$$\left[\begin{array}{cc} \mathbf{A} & \mathbf{B} \\ \mathbf{C} & \mathbf{D} \end{array}\right]^{-1} = \left[\begin{array}{cc} \mathbf{A}^{-1}+\mathbf{A}^{-1}\mathbf{B}(\mathbf{D}-\mathbf{C}\mathbf{A}^{-1}\mathbf{B})^{-1}\mathbf{C}\mathbf{A}^{-1} & -\mathbf{A}^{-1}\mathbf{B}(\mathbf{D}-\mathbf{C}\mathbf{A}^{-1}\mathbf{B})^{-1} \\ -(\mathbf{D}-\mathbf{C}\mathbf{A}^{-1}\mathbf{B})^{-1}\mathbf{C}\mathbf{A}^{-1} & (\mathbf{D}-\mathbf{C}\mathbf{A}^{-1}\mathbf{B})^{-1} \end{array}\right] .$$

Let $\mathbf{X}$ be the design matrix that collects the $\underline{x}_i$'s as its rows, $\mathbf{X} = [\mathbf{X}_r \;\; \mathbf{X}_J]$ be its split into the parts corresponding to the "reduced model" and the predictors $J$ under examination, and

$$\begin{aligned} \mathbf{K}_r &= \left(\mathbf{X}_r^\top \mathbf{X}_r\right)^{-1} , \qquad \mathbf{H}_r = \mathbf{X}_r\mathbf{K}_r\mathbf{X}_r^\top \\ \mathbf{M}^{-1} &= \mathbf{X}_J^\top(\mathbf{I}-\mathbf{H}_r)\mathbf{X}_J , \qquad \mathbf{G} = \mathbf{X}_J\mathbf{M}\mathbf{X}_J^\top . \end{aligned}$$

Inverting $\mathbf{X}^\top\mathbf{X}$ then leads to

$$\left(\mathbf{X}^\top\mathbf{X}\right)^{-1} = \left[\begin{array}{cc} \mathbf{K}_r+\mathbf{K}_r\mathbf{X}_r^\top\mathbf{G}\mathbf{X}_r\mathbf{K}_r & -\mathbf{K}_r\mathbf{X}_r^\top\mathbf{X}_J\mathbf{M} \\ -\mathbf{M}\mathbf{X}_J^\top\mathbf{X}_r\mathbf{K}_r & \mathbf{M} \end{array}\right] . \qquad (1)$$

It is well known that

$$\mathrm{var}(\widehat{\underline{\beta}}_J) = \sigma^2\Big(\left(\mathbf{X}^\top\mathbf{X}\right)^{-1}\Big)_{JJ} = \sigma^2\mathbf{M} .$$

Since $\mathbf{C} = \frac{1}{n}\mathbf{X}^\top\mathbf{X}$,

$$\begin{aligned} p\,\eta_J^2 &= (\underline{\beta}_J-\underline{\vartheta}_0)^\top\left(\left(\mathbf{C}^{-1}\right)_{JJ}\right)^{-1}(\underline{\beta}_J-\underline{\vartheta}_0)/\sigma^2 \\ &= (\underline{\beta}_J-\underline{\vartheta}_0)^\top\Big(n\,\mathrm{var}(\widehat{\underline{\beta}}_J)\Big)^{-1}(\underline{\beta}_J-\underline{\vartheta}_0) , \end{aligned}$$

and in this sense, $\eta_j$ is the norm of a standardized effect.

Assume that there is an intercept $\beta_0$ in the model. Let $J$ collect the remaining coefficients, $J = \{j|j\neq 0\}$. In order to apply equation (1), note that $\mathbf{K}_r = (\underline{1}^\top\underline{1})^{-1} = 1/n$, $\mathbf{H}_r = \underline{1}\underline{1}^\top/n$,

$$\mathbf{M}_{JJ}^{-1} = \mathbf{X}_J^\top\left(\mathbf{I}-\underline{1}\underline{1}^\top/n\right)\mathbf{X}_J = \mathbf{X}_J^\top\mathbf{X}_J - \overline{\underline{X}}_J\overline{\underline{X}}_J^\top n = \mathbf{X}_c^\top\mathbf{X}_c ,$$

where $\overline{\underline{X}}_J$ is the vector of column averages of $\mathbf{X}_J$ and $\mathbf{X}_c$ is the matrix of centered columns, $\mathbf{X}_c = \mathbf{X}_J - \underline{1}\,\overline{\underline{X}}_J^\top$. Therefore,

$$\Big(\left(\mathbf{X}^\top\mathbf{X}\right)^{-1}\Big)_{JJ} = \left(\mathbf{X}_c^\top\mathbf{X}_c\right)^{-1} ,$$

and inference about $\underline{\beta}_J$ can be calculated on the basis of the centered design matrix—a well known result. Applying equation (1) again with a single $j$ to the centered $\mathbf{X}_c$ shows

$$\mathbf{M}^{-1} = \underline{X}_c^{(j)\top}(\mathbf{I}-\mathbf{H}_r)\underline{X}_c^{(j)} = ||\underline{X}_c^{(j)}||^2(1-R_j^2) = (n-1)s_j^2(1-R_j^2)$$

where $R_j$ is the multiple correlation between $\underline{X}^{(j)}$ and the other predictors, $\mathbf{X}_r$, and therefore,

$$\vartheta_{\mathsf{drop},j}^2 = \beta_j^2\Big/\Big(n\,\mathrm{var}\Big(\widehat{\beta}_j\Big)\Big) = \beta_j^2\big/\left(n\mathbf{M}\sigma^2\right) = \beta_j^2 s_j^2\left(1-R_j^2\right)/\sigma^2 = \vartheta_j^2\kappa_n^2\left(1-R_j^2\right) .$$
