# Supplementary material for: New relevance and significance measures to replace p-values
Source: PLoS One. 2021 Jun 16;16(6):e0252991. doi: 10.1371/journal.pone.0252991 (PMC8208587; doi:10.1371/journal.pone.0252991)
Supplement: Attachment — Submitted filename: reply-reviewer.pdf [file pone.0252991.s002.pdf]

Measuring significance and relevance instead of p-values: Reply to the Reviewer

Werner A. Stahel

January 1, 2021

Dear Reviewer Thank you for your positive evaluation and your helpful comments. Here are my reactions to them.

Section 4 offers guidance on selecting a threshold of relevance. The author proposes to quantify the threshold using a formulaic approach. Any such quantity is arbitrary and wont be applicable across all situations. This is illustrated in lines 495-499 where the author points to a situation in which the proposed threshold does not work and notes that the scientific question should guide the choice of the effect scale and of the relevance threshold! The author returns to this point in lines 596-598, and explains that he offered the formulaic approach because conventions facilitate such a burden, it is foreseeable that they will be invented and adhered to sooner or later therefore, considerations for such choices have been introduced in this paper. It would be helpful if the author would provide that rationale at the beginning of Section 4, rather than leaving it to Section 6.

A good point. I have adjusted the paragraphs concerned.

Section 5 offers guidance on reporting results. Here the simplicity of the approach is often lost, as the guidance suggests reporting values for a good number of items. Why not report the estimated parameter, its RLe, and its CI? The author makes the same argument in lines 608-609, but that is not what he has presented, for example, in Table 7.

The simple description by estimated effect and respective Rls has been introduced at the beginning of Section 5 and given in the text for every example. For the regression example, an extensive table is presented (Table 7). The use of the more important indicators, shown now in bold face, is now explained in the text.

Table 1. s and r are used in every row in the table but are undefined. If s a placeholder for Sig and r a placeholder for RL, it would have been clearer to use the appropriate column heading in place of s or r in the body of the table.

Yes, these are place holders. I prefer to add this explanation in the table caption, but could agree with your suggestion if you insist.

Line 226. I believe there is an error in using theta.

Yes, thank you for the hint.

... and for your positive review with the helpful constructive comments as a whole.

Best regards

Werner Stahel
