# Supplementary material for: New relevance and significance measures to replace p-values
Source: PLoS One. 2021 Jun 16;16(6):e0252991. doi: 10.1371/journal.pone.0252991 (PMC8208587; doi:10.1371/journal.pone.0252991)
Supplement: Attachment — Submitted filename: stahel.pdf [file pone.0252991.s003.pdf]

Referee report on New relevance and significance measures to replace p-values

Werner A. Stahel

February 24, 2021

This is an interesting paper with a new proposal to replace p-values by other measures of significance an relevance. The main idea is to define a threshold of relevance and deduce a t-type statistics to have a single value. I do agree with the author, that is useful to have, because people often like to have one value (like the p-value) to make a decision about their study. This easy measure is similar to a t-value for a one sided test for $\theta >= \xi$, but by multiplying with the respective quantile of the t- distribution (or normal distribution) one gets a decision limit of 1, which makes it attractive. Furthermore, the author provides an R-package for calculating the relevant measures in simple cases. I think this main idea should be published, but I have some serious doubts whether the more complex cases the author mentions, are addressed in an adequate way. So I propose to publish the nice main ides of the paper and leave out some of the more complex models, which are not in the R package and need a more detailed elaboration. Furthermore a discussion of the Bayesian method should be made, see my comments below

My major points are:

1. The authors statememnt *Bayesian methods have also been advertized as a methodology that gives a more differentiated picture. None of these proposals have yet been widely applied* is not acceptable. The author should discuss the Bayesian approach and proposals. A simple analogue to the measure proposed by the author is then posterior probability that $\theta > \xi$. Furthermore, the frequentistic interpretation of a confidence interval and also of the measures proposed in the paper should be discussed.

2. Chapter 3: Since the basic method is one dimensional and it cannot be generalized easily for the multivariate case, I would describe the very important case of the two sample problem with a one dimensional

parameter θ. The the relevance can be described by the usual t- test (please use the realistic setting of the t -Test for unequal variances). The discussion of using strategies like Cohen d causes some confusion, since this is a problem of defining a relevance threshold and not of the method proposed in the paper. Therefore, the discussion should be made in Chapter 4.

3. Chapter 3.2 Proportions: I would prefer to call the chapter *probabilities* or *binary variables*. I would omit the variance stabilizing transformations and concentrate on risks (exact binomial test) and (log) odds ratios. In my view the Fischer test and relating exact confidence intervals for odds ratios is the suitable approach avoiding transformations.

4. Chapter 3.3. Again the discussion about scaling of the effect should done in Chapter 4. The new method can be illustrated by using a the regression parameter β with a relevance threshold from substantive reasoning.

5. multiple regression: Her also the new method could be clearly illustrated by using a t- test for a regression parameter avoiding the discussion of scaling.

6. Random design. In the context of predictions, significance tests are not used. There is a huge literature of variable importance in the literature of statistical learning and machine learning using e.g. cross validation techniques. I think the approach by the author is not yet mature and is beyond the scope of the paper.

7. The approach for the multivariate case is not intuitive and could be omitted. The main point is that the covariance structure of the observed data does not translate to intuitive areas. So a better alternative is to consider the components separately with the methods proposed in Chapter 2.

8. For generalized linear models $E(Y|x) = G[x^T\beta)$ (logistic, poisson etc.), the method proposed by the author can be directly applied for each covariate using the confidence interval for the each parameter β from the asymptotic normal distribution. In logistic regression a relevant effect could be given by a threshold $\exp(\beta)$ for the odds ratio.
