# Supplementary material for: New relevance and significance measures to replace p-values
Source: PLoS One. 2021 Jun 16;16(6):e0252991. doi: 10.1371/journal.pone.0252991 (PMC8208587; doi:10.1371/journal.pone.0252991)
Supplement: Attachment — Submitted filename: refrep-response2105.pdf [file pone.0252991.s004.pdf]

“New relevance and significance measures to replace p-values” Response to the Referee’s Comments

> *This is an interesting paper with a new proposal to replace p-values by other measures of significance an relevance. The main idea is to define a threshold of relevance and deduce a t-type statistics to have a single value...*

Thank you for the positive overall appreciation of the main idea. I have the impression that your description characterizes the significance measure more closely than the relevance, whereas the latter is in the focus of the paper.

> *I have some serious doubts whether the more complex cases the author mentions, are addressed in an adequate way. So I propose to publish the nice main ides of the paper and leave out some of the more complex models, which are not in the R package and need a more detailed elaboration.*

My goal is to cover the important models that appear frequently in applications in order to make the concepts directly applicable. I think that it is important to show that these are general concepts that apply directly to the practicle situations. Often, concepts in this area are introduced for the simplest case(s) only and generalization needs further development, and they therefore remain theoretical.

I have tried to make the paper more readable by collecting the more technical case of regression into a seperate section, which may be left out in a first run. But there is an important aspect of the whole line of thought, which comes out in this context very clearly: The type of scientific question should guide the choice of the method—and in our theme the type of effect measure—to be calculated and interpreted. Thus, it would seriously reduce the value of the paper if this section was cut out.

Nevertheless, this comment was very useful since it lead me to clarify the development of the concept of relevance and establish very clearly that it is a parameter of the model, defined independently of the way it is estimated. This also avoids some controversial points about estimation that you rise below.

> *Furthermore a discussion of the Bayesian method should be made, see my comments below.*

1. The author states “Bayesian methods have also been advertized as a methodology that gives a more differentiated picture. None of these proposals have yet been widely applied is not acceptable.” The author should discuss the Bayesian approach and proposals. A simple analogue to the measure proposed by the author is then posterior probability that $\theta > \xi$. Furthermore, the frequentistic interpretation of a confidence interval and also of the measures proposed in the paper should be discussed.

The ideas to consider a relevance threshold and specifically to consider the posterior probability that $\theta > \xi$ seem to be new in the Bayesian literature. If they have appeared, I would be grateful to get references, which I would gladly mention in my paper.

Nevertheless, I have been pointed to a Bayesian paper that is rather closely related to the concept of relevance: “Rejecting or Accepting Parameter Values in Bayesian Estimation” by

John Kruschke, and I gladly discuss this briefly in the Introduction and in the subsection on "Classification of results."

> *2. Chapter 3: Since the basic method is one dimensional and it cannot be generalized easily for the multivariate case, I would describe the very important case of the two sample problem with a one dimensional parameter θ.*

The two sample problem is discussed now in Section 3.1 before the general model is introduced. This is one of the improvements of the exposition that should make the paper more easily accessible, as described above.

> *The the relevance can be described by the usual t-test (please use the realistic setting of the t-test for unequal variances).*

The relevance relates the effect parameter to be estimated to the threshold. There is then an estimated relevance with a confidence interval. The value that is recommended to be in the focus cannot be expressed simply using just the t statistic. I therefore fail to understand this argument.

This is one of the technical details that can be avoided, since estimation of the *parameter* relevance can be done in the appropriate way in an application.

> *The discussion of using strategies like Cohen d causes some confusion, since this is a problem of defining a relevance threshold and not of the method proposed in the paper. Therefore, the discussion should be made in Chapter 4 (now 5).*

The paper more clearly distigushes between the "effect scale" and the threshold. Cohen's d would be a suitable choice of effect scale, but I prefer $\vartheta = d/2$ for reasons of consistency with other situations. Your point helped describe this aspect.

> *3. Chapter 3.2 Proportions: I would prefer to call the chapter probabilities or binary variables. I would omit the variance stabilizing transformations and concentrate on risks (exact binomial test) and (log) odds ratios. In my view the Fischer test and relating exact confidence intervals for odds ratios is the suitable approach avoiding transformations.*

I have followed your recommendation and dropped the variance stabilizing transformation, and I indeed emphazise log odds—the preferrable transformation. Estimation is not explicitly mentioned any more.

I still prefer the title "Proportions" because these can be expressed as probabilities as well as odds, they can be compared by odds ratios, and the section applies to binary as well as binomial data.

> *4. Chapter 3.3. Again the discussion about scaling of the effect should done in Chapter 4 (now 5). The new method can be illustrated by using the regression parameter β with a relevance threshold from substantive reasoning.*

For the slope parameter in simple regression, the effect scale is obtained by a linear transformation and therefore less essential. It is still suitable for making effects comparable between studies. For the correlation coefficient, the transformation is nonlinear and achieves a kind of comparability between differences around 0 and towards the extreme values -1 and

+1. Thus, the argument given above applies.

> *5. multiple regression: Here also the new method could be clearly illustrated by using a t-test for a regression parameter avoiding the discussion of scaling.*

See comment to 4.

> *6. Random design. In the context of predictions, significance tests are not used. There is a huge literature of variable importance in the literature of statistical learning and machine learning using e.g. cross validation techniques. I think the approach by the author is not yet mature and is beyond the scope of the paper.*

Yes, "variable importance" is a concept with many ways of formalization. From a point of view of asking a scientific question about forecasting, it is natural to choose the precision of forecasts as the important effect parameter. The discussion of this point in the paper shows in a nutshell that the choice of a method should reflect the nature of the scientific question to be studied. If the prupose was varianble selection as a step of model development, other criteria may prove more useful. Cross validation is not a criterion in itself, but a method to estimate a given criterion with minimal assumptions about the model.

> *7. The approach for the multivariate case is not intuitive and could be omitted. The main point is that the covariance structure of the observed data does not translate to intuitive areas. So a better alternative is to consider the components separately with the methods proposed in Chapter 2.*

The proposed extention does not rely on "covariance structure of the observed data", but has been defined in terms of the covariance of the estimated parameter vector. If is now based on the Fisher information, which is a property of the model and no longer depends on the estimation method—although it reflects the covariance of the maximum likelihood estimator.

The idea of examining the components separately is certainly often another plausible way to treat a problem (as mentioned in the paper). However, in the case of regression with a factor as explanatory variable, the individual regression coefficients of the technical variables expressing the chosen contrasts are not generally interpretable in a sensible way. Thus, this rather important case would remain without a recommended method if the multivariate case were dropped.

> *8. For generalized linear models $E(Y|x) = G(x^T\beta)$ (logistic, poisson etc.), the method proposed by the author can be directly applied for each covariate using the confidence interval for the each parameter β from the asymptotic normal distribution. In logistic regression a relevant effect could be given by a threshold $\exp(\beta)$ for the odds ratio.*

Again, the confidence interval is not enough.

In summary, your comments have been very helpful to guide me in a thorough revision of the paper. I hope that you will be more happy with this new version.
